# Infection and transmission of Cache Valley virus by *Aedes albopictus* and *Aedes aegypti* mosquitoes

**DOI:** 10.1186/s13071-019-3643-0

**Published:** 2019-07-31

**Authors:** Victoria B. Ayers, Yan-Jang S. Huang, Amy C. Lyons, So Lee Park, James I. Dunlop, Isik Unlu, Alain Kohl, Stephen Higgs, Bradley J. Blitvich, Dana L. Vanlandingham

**Affiliations:** 1 Department of Diagnostic Medicine/Pathobiology, College of Veterinary Medicine, Kansas State University, Manhattan, KS 66506 USA; 2 Biosecurity Research Institute, Kansas State University, Manhattan, KS 66506 USA; 3 Centre for Virus Research, MRC-University of Glasgow, Glasgow, G61 1QH Scotland, UK; 4 Broward County Mosquito Control, Pembroke Pines, FL 33023 USA; 5 Center for Vector Biology, Rutgers University, New Brunswick, NJ 08901 USA; 6 Department of Veterinary Microbiology and Preventive Medicine, College of Veterinary Medicine, Iowa State University, Ames, IA 50011 USA

**Keywords:** Cache Valley virus, *Aedes albopictus*, *Aedes aegypti*, Competent vectors

## Abstract

**Background:**

Cache Valley virus (CVV; *Bunyavirales*, *Peribunyaviridae*) is a mosquito-borne arbovirus endemic in North America. Although severe diseases are mainly observed in pregnant ruminants, CVV has also been recognized as a zoonotic pathogen that can cause fatal encephalitis in humans. Human exposures to CVV and its related subtypes occur frequently under different ecological conditions in the New World; however, neurotropic disease is rarely reported. High prevalence rates of neutralizing antibodies have been detected among residents in several Latin American cities. However, zoophilic mosquito species involved in the enzootic transmission are unlikely to be responsible for the transmission leading to human exposures to CVV. Mechanisms that lead to frequent human exposures to CVV remain largely unknown. In this study, competence of two anthropophilic mosquitoes, *Aedes albopictus* and *Ae. aegypti*, for CVV was determined using *per os* infection to determine if these species could play a role in the transmission of CVV in the domestic and peridomestic settings of urban and suburban areas.

**Results:**

*Aedes albopictus* were highly susceptible to CVV whereas infection of *Ae. aegypti* occurred at a significantly lower frequency. Whilst the dissemination rates of CVV were comparable in the two species, the relatively long period to attain maximal infectious titer in *Ae. aegypti* demonstrated a significant difference in the replication kinetics of CVV in these species. Detection of viral RNA in saliva suggests that both *Ae. albopictus* and *Ae. aegypti* are competent vectors for CVV under laboratory conditions.

**Conclusions:**

Differential susceptibility to CVV was observed in *Ae. albopictus* and *Ae. aegypti*, reflecting their relatively different capacities for vectoring CVV in nature. The high susceptibility of *Ae. albopictus* to CVV observed in this study suggests its potential role as an efficient vector for CVV. Complemented by the reports of multiple CVV isolates derived from *Ae. albopictus*, our finding provides the basis for how the dispersal of *Ae. albopictus* across the New World may have a significant impact on the transmission and ecology of CVV.

## Background

Cache Valley virus (CVV) is a mosquito-borne arbovirus (genus *Orthobunyavirus*, family *Peribunyaviridae*). The virus was first isolated from *Culiseta inornata* in Utah, USA, in 1956 [1], and is now regarded as the most widely distributed member within the Bunyamwera serogroup in the New World [2]. CVV has been found extensively throughout North America with several known regional subtypes, including Maguari virus, Xingu virus, and Fort Sherman virus circulating in Latin America [3–6]. Historically, CVV has been regarded as an important agricultural pathogen associated with embryonic lethality and abortions in ruminants. Infections in humans are largely asymptomatic or associated with mild febrile diseases. Although the association between CVV infection and neurological human diseases was first suggested in 1995, the pathogenicity of CVV in humans was not recognized until several cases of neurotropic diseases were directly attributed to CVV infection [7]. As an endemic pathogen in the Americas, serological surveys have demonstrated that humans in the New World can be exposed to CVV under various ecological conditions. The intensive transmission of CVV on the Eastern Shore of Maryland and Virginia, USA, coincided with the high seroprevalence rate among residents of Chincoteague Island, where saltwater marsh is the predominant mosquito habitat [8, 9]. Based on serology, CVV was demonstrated to occur in the urban and suburban environments in Latin America, where the majority of mosquito infestation is associated with container-inhabiting mosquitoes, especially *Aedes aegypti* and *Ae. albopictus* [10–12]. In the capital city of Mérida in the Yucatan State, Mexico, neutralizing antibodies against CVV can be found in 18% of individuals with febrile illness [9]. Similarly, the silent transmission of CVV also led to up to 8% seroprevalence rate among residents of the Córodoba Province, Argentina [13]. Despite the evidence suggesting frequent transmission of CVV to humans across the Americas, very little is known about the specific vectors responsible for transmission to humans. A major limitation in our understanding of mechanisms responsible for the high prevalence rates of neutralizing human antibodies in the urban and suburban areas is the lack of knowledge on the vector competence of domestic and peridomestic mosquitoes for CVV.

CVV has been isolated from over 30 species of mosquitoes [14]. However, the majority of competent vectors for CVV are not domestic or peridomestic species that can efficiently spread arboviruses among humans in the cities. For instance, the two endemic vectors for CVV, *Anopheles quadrimaculatus* and *Culiseta inornata* are not common in urban and suburban areas [15, 16]. Other competent species, including *Ae. taeniorhynchus* and *Ae. sollicitans*, are normally found in saltwater marshes [17, 18]. As a potential bridge vector that is known to support the transmission of CVV, the distribution of *Culex tarsalis* is rare under urban landscapes and precludes its potential role as a domestic or peridomestic vector [19, 20]. Domestic *Culex* spp. mosquitoes which contributed to the isolation of CVV in nature are known to be refractory through *per os* infection, suggesting that CVV in the urban and suburban areas are likely to be vectored by other mammophillic species [20].

Isolation of CVV from *Ae. albopictus* and *Ae. japonicus* is suggestive of the potential involvement of domestic and peridomestic *Aedes* species mosquitoes in the transmission of CVV from animals to humans [21, 22]. In the northeastern USA, both species have been found to be mammophillic, feeding on humans and white-tailed deer, a major amplification host of CVV [23–25]. With the exception of *Ae. japonicus*, which has previously been investigated for its competence for CVV under laboratory conditions, the vectorial efficiency of domestic and peridomestic *Aedes* species for CVV remains largely undetermined [26]. In this study, *Ae. albopictus* and *Ae. aegypti* were orally challenged with CVV to investigate the dynamics of infection, dissemination and transmission. The results demonstrate that *Ae. albopictus* can be an efficient vector for CVV and provide the basis of our knowledge in the transmission of CVV to humans in the urban and suburban environment.

## Methods

### Virus and *per os* challenge of mosquitoes

The prototype CVV 6V633 strain was used in all oral challenge experiments. Virus stocks were propagated and titered in African green monkey kidney Vero76 cells in Leibovitz’s L-15 media (Thermo Fisher Scientific, Waltham, MA, USA) supplemented with 10% fetal bovine serum (Thermo Fisher Scientific, Waltham, MA, USA), 10% tryptose phosphate broth (Sigma-Aldrich, St. Louis, MO, USA), penicillin/streptomycin (Thermo Fisher Scientific, Waltham, MA, USA) and l-glutamine (Thermo Fisher Scientific, Waltham, MA, USA), as previously described [20]. Frozen stocks at 7.95 log median tissue culture infectious dose (TCID_50_)/ml were thawed at 37 °C and mixed with an equal volume of defibrinated sheep blood (Colorado Serum Company, Denver, CO, USA) to generate infectious blood meals.

*Per os* infection of mosquitoes was performed with 7-to-10-day-old female *Ae. aegypti* Higgs white-eye strain (F > 20), and with *Ae. albopictus* (F_4_), which were derived from eggs collected from the city of Trenton, Mercer County, NJ, USA, in July 2016. In each of the three independent repeats of oral challenge experiments, 2 cartons of 100 mosquitoes each were orally exposed to viremic blood meals containing CVV. Mosquitoes were deprived of sucrose for 24 h prior to infection. Infectious blood meals were administered through a Hemotek membrane feeding system (Discovery Workshop, Lancashire, United Kingdom) using previously described techniques [27]. Engorged mosquitoes were cold-anesthetized, collected, and maintained at 28 °C for 14 days as previously described [10]. Up to three engorged mosquitoes at the end of each oral challenge experiment were collected and titrated to confirm the ingestion of infectious viruses. Orally challenged mosquitoes were mechanically aspirated at 7 and 14 days post-infection (dpi) for the assessment of infection status. Forced salivation of immobilized mosquitoes was also performed at 14 dpi to determine the incidence of transmission [20].

### Detection of infectious virus

The dynamics of infection and dissemination was determined based on the detection of infectious viruses in homogenized tissues dissected from mosquitoes collected at 7 and 14 dpi. Whole mosquitoes were also collected without dissection to assess the growth kinetics of CVV in infected mosquitoes. Samples were homogenized at 26 Hz for 4 min and titrated using Vero76 cells. All concentrations of infectious viruses are calculated as TCID_50_/ml [5]. Reverse-transcriptase nested polymerase chain reactions were performed to detect viral RNA present in mosquito saliva. Viral RNA was extracted with QIAamp Viral RNA Mini Kit (Qiagen, Valencia, CA, USA), reverse transcribed with Superscript III reverse transcriptase (Invitrogen, Carlsbad, CA, USA) and amplified using Platinum Taq DNA polymerase (Thermo Fisher Scientific, Waltham, MA, USA) as previously described [20]. A gene-specific primer (CV-Mex-R: 5′-GAC GTC TGT TAA GAA GCA AGT TGA GTT T-3′) was used for cDNA synthesis followed by the amplification using nested PCR (first primer set: CV-Mex-F: 5′-GCA CTC TGG CAG GCA GGA-3′ and CV-Mex-R: 5′-GAC GTC TGT TAA GAA GCA AGT TGA GTT T-3′; second primer set: CV-G1-F: 5′-CCA ATG CAA TTC AGG GCA GT-3′ and CV-G1-R: 5′-TGA GTC ACC ACA TGC TGT AAG GT-3′). All amplicons were separated and visualized by electrophoresis on 4% agarose gels at 125V for 40 min.

### Statistical analysis

Infection, dissemination, and transmission rates of CVV were calculated based on the criteria previously described [20]. Briefly, infection rates of CVV were calculated based on the incidence for the positive detection of infectious viruses in both dissected mosquitoes and whole mosquito carcasses. Dissemination rates were determined based on the percentage of positive virus isolation from the secondary tissues of dissected mosquitoes that were infected with CVV. Differences in the percentage of infection, dissemination, and transmission were determined using Chi-square test or Fisher’s exact test, depending on the sample sizes in the contingency tables. Titers of infected mosquitoes were compared with Mann-Whitney rank sum test between two groups when normal distribution is not observed or Student’s t-test when infectious titers follow normal distribution. All statistical analyses were conducted using GraphPad (San Diego, CA, USA), SigmaPlot (San Jose, CA, USA), and Excel software (Redmond, WA, USA).

## Results

### Differential susceptibility to CVV between *Ae. albopictus* and *Ae. aegypti*

**Fig. 1:**
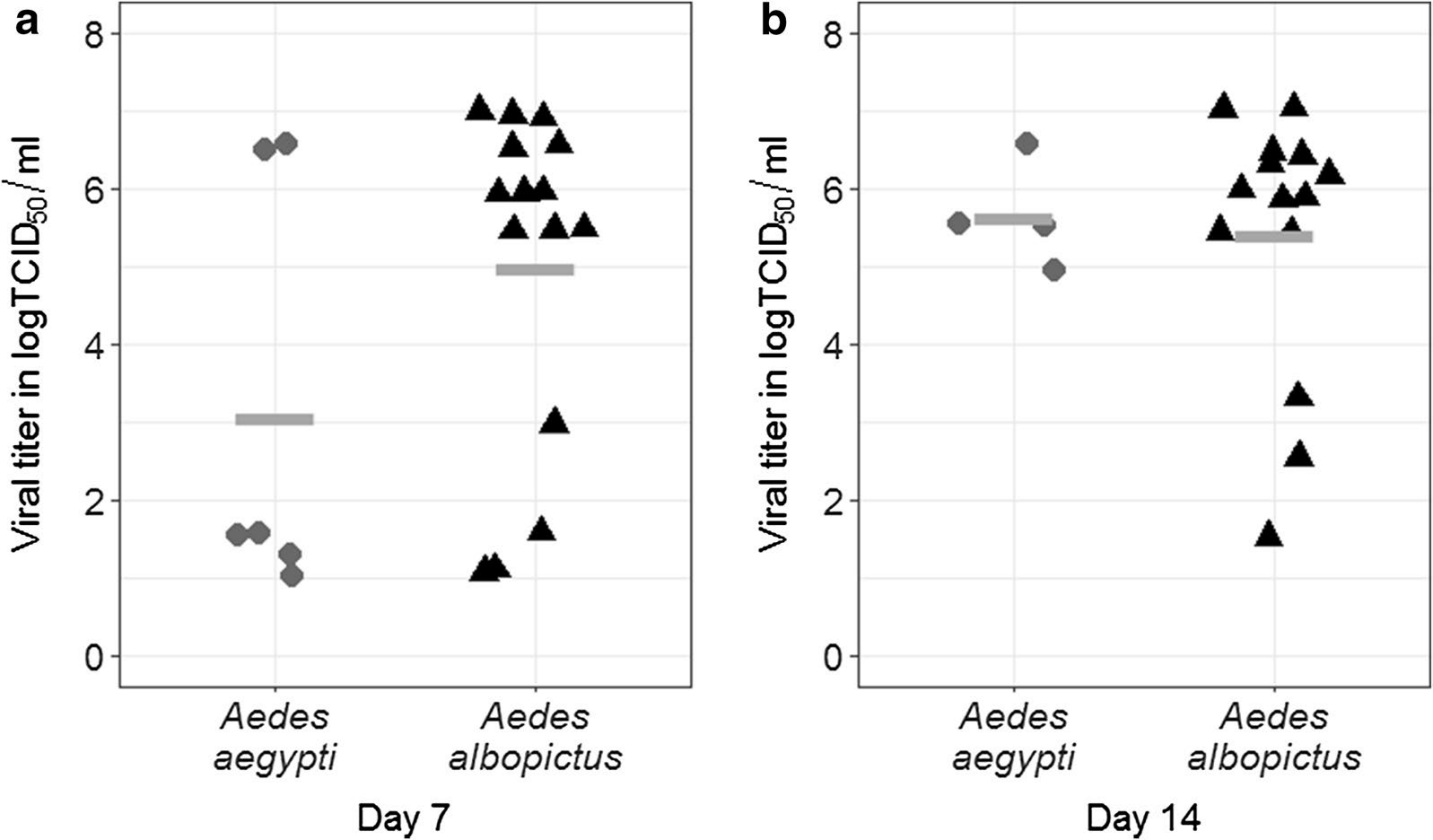
Infectious titers of whole mosquitoes infected with CVV at 7 and 14 dpi. Titers of individual infected *Ae. aegypti* and *Ae. albopictus* are shown in circle and triangle, respectively. The grey solid line represents the average titer of each species at 7 and 14 dpi

Oral challenge with CVV led to the establishment of infection in both *Ae. albopictus* and *Ae. aegypti*. As summarized in Table 1, the significantly higher infection rates were observed in *Ae. albopictus* at both 7 dpi [*Ae. albopictus:* 69.2% (45/65) *vs Ae. aegypti*:15.2% (10/66), Chi-square test: *χ*^2^ = 37.13, *df* = 1, *P *< 0.001] and 14 dpi [*Ae. albopictus:* 56.5% (26/46) *vs Ae. aegypti*: 11.0% (9/82), Chi-square test: *χ*^2^ = 28.52, *df* = 1, *P *< 0.001], indicating higher susceptibility of *Ae. albopictus* to CVV than *Ae. aegypti*. Consistent with the differences in susceptibility, *Ae. albopictus* supported more rapid replication of CVV as demonstrated by significantly higher average titers of whole mosquitoes collected at 7 dpi [*Ae. albopictus* (average titer ± standard deviation): 5.0 ± 2.2 logTCID_50_/ml *vs Ae. aegypti* (average titer ± standard deviation): 3.1 ± 2.7 logTCID_50_/ml; t-test: *t* = 1.713, *df* = 19; *P* = 0.02] (Fig. 1). Although there was no significant difference in titers of infected mosquitoes at 14 dpi [*Ae. albopictus* (median titer): 6.0 logTCID_50_/ml *vs Ae. aegypti* (median titer): 5.5 logTCID_50_/ml; Mann–Whitney test: *U* = 22, *P* = 0.55].

**Table 1:** Average titers of engorged mosquitoes, infection, dissemination, and transmission rates in mosquitoes challenged with CVV

Mosquito species	0 dpi	7 dpi		14 dpi		
	Average titers of engorged mosquitoes (logTCID_50_/ml)	Infection rate (%)^ab^	Dissemination rate (%)^c^	Infection rate (%)^ab^	Dissemination rate (%)^c^	Transmission rate (%)^d^
*Ae. albopictus*	3.7 ± 0.6 (*n* = 11)	69.2 (45/65)	83.3 (25/30)	56.5 (26/46)	100.0 (12/12)	29.6 (8/27)
*Ae. aegypti*	4.0 ± 0.8 (*n*=18)	15.2 (10/66)	100 (4/4)	11.0 (9/82)	100 (5/5)	30.0 (3/10)

^a^The infection rate of CVV at 7 and 14 dpi was determined by the isolation of infectious viruses in tissues of dissected mosquitoes or carcasses of whole mosquitoes using Vero76 cells

^b^Significant differences between *Ae. albopictus* and *Ae. aegypti* were detected using Chi-square test

^c^The dissemination rate of CVV at 7 and 14 dpi was calculated based on the detection of infectious viruses in secondary tissues (head, wings and legs) of dissected mosquitoes, which were infected with CVV

^d^The transmission rate of CVV was determined by the incidence of positive detection of viral RNA among saliva of infected mosquitoes using nested RT-PCR

Whilst significant differences in susceptibility and replication kinetics were observed, the incidence of disseminated infection was indistinguishable at 7 dpi [*Ae. albopictus:* 83.3% (25/30) *vs Ae. aegypti*: 100% (4/4), Fisher’s exact test: *χ*^2^ = 37.13, *df* = 1, *P* = 1.00] and 14 dpi [*Ae. albopictus*: 100% (12/12) *vs Ae. aegypti*: 100% (5/5)]. Our results indicate that infection with CVV can be established in both *Ae. albopictus* and *Ae. aegypti* through the ingestion of viremic blood meals.

### Domestic and peridomestic *Aedes* species mosquitoes are competent vectors for CVV

At 14 dpi, viral RNA was detected in saliva collected from 29.6% (8/27) of infected *Ae. albopictus*. Similarly, 30.0% (3/10) of infected *Ae. aegypti* also showed a positive detection of viral RNA in the saliva. The presence of viral RNA following oral exposure to CVV indicates that both species are competent vectors for CVV. Although the transmission rate for both species was approximately 30%, the higher infection rates for *Ae. albopictus*, as compared to *Ae. aegypti*, indicate that there could potentially be more infected *Ae. albopictus* involved in the overall transmission of CVV.

## Discussion

The results of our study demonstrated that both *Ae. albopictus* and *Ae. aegypti* are susceptible to CVV. The differential susceptibility suggests there can potentially be a difference in the likelihood of the two species in vectoring CVV to humans in nature, especially in the southern USA and Latin America. The relatively high competence of *Ae. albopictus* demonstrated in this study, and large numbers of isolates recovered from nature, suggest that this species may be actively involved in the enzootic and epizootic transmission of CVV in regions where viremic vertebrate hosts and humans coexist [22]. Interestingly, detection of CVV in *Ae. albopictus* coincides with the dispersal of lineage 2 viruses from southern Mexico to the northeastern USA. All CVV isolates from *Ae. albopictus* in northeastern USA have also been demonstrated to cluster under the same lineage 2. These findings warrant further investigation whether the species also contributed to the emergence of the new genetic lineage in North and Central America [22, 28]. As the distribution of *Ae. aegypti* in North America has just exceeded 33°N latitude between 2011 and 2014 and the introduction of *Ae. albopictus* did not take place until the 1980s, the high prevalence of neutralizing antibodies against CVV in the eastern shore of Maryland and Virginia observed in the 1960s was unlikely to be caused by transmission vectored by the two competent species identified in this study [8]. A more plausible explanation might be the involvement of other vectors known for zoonotic transmission of arboviruses including *Ae. sollicitans* and *Ae. taeniorhynchus*.

As an invasive species, the increasing infestation of *Ae. albopictus*, and its high competence for CVV, also raised an interesting question: whether or not the introduction and potential spread of this species will change the epidemiology of CVV and other agriculturally important arboviruses in different regions of the Americas [29]? Increased autochthonous transmission of various human and zoonotic arboviruses vectored by *Ae. albopictus* has established its importance as a species that impacts human public health [30]. However, much less is known regarding its importance with respect to animal health. It will be of great human and animal health importance to further define the role of *Ae. albopictus* in vectoring CVV among animal reservoirs and humans, especially those located in infested areas. The findings may be helpful in defining the health risk associated with CVV infection, which remains largely unknown.

Based on our results, *Ae. aegypti* is likely to have limited contribution to transmission of CVV in nature because of the low susceptibility demonstrated in this study. The use of *Ae. aegypti* Higgs white-eye strain, a colonized strain derived from the Puerto Rican RexD colony and selected based on the high competence of a variety of arboviruses including several orthobunyaviruses, further support our conclusion [31]. Although the species can be competent for CVV under laboratory conditions, a large number of infected mosquitoes may be required for the intensive transmission that leads to the observed occurrence of a high seroprevalence rate. Entomological surveys have demonstrated that naturally occurring infection from *Ae. aegypti* with CVV is a rare event [32]. Therefore, *Ae. aegypti* is unlikely to serve as an important urban vector responsible for frequent human exposures to CVV and its related subtypes. To the best of our knowledge, infestation of *Ae. albopictus* has not yet been reported in the Córodoba province, Argentina. The population of *Ae. aegypti* has been known to be involved in the transmission of arboviruses in the region but should not contribute to the transmission of CVV to humans [33, 34]. Collectively, available evidence suggests that high prevalence rates of human neutralizing antibodies against CVV in Latin America may involve transmission by other mammophillic domestic and peridomestic mosquito species. Identifying such species will be particularly important for the advancement of our knowledge for the ecology of CVV and other regional subtypes in selected regions in Latin America.

## Conclusions

For the first time, our laboratory investigation suggests that two *Aedes* species known for their competence of important pathogenic arboviruses such as dengue and yellow fever viruses, are also competent for CVV. The differential susceptibility between *Ae. albopictus* and *Ae. aegypti* indicates the potential difference in the efficiency of vectoring CVV to humans in nature. As a highly susceptible species competent for the transmission of CVV, populations of *Ae. albopictus*, which feed on viremic hosts and humans, can be of significance in veterinary public health and the ecology of CVV and its related subtypes in the Americas, as the infestation of *Ae. albopictus* continues to be reported in different regions.

## Data Availability

Data generated in this study are available from the corresponding authors upon reasonable request.

## Abbreviations

CVV: Cache Valley virus
dpi: days post infection
TCID_50_: median tissue culture infectious dose
